# *Lama guanicoe* remains from the *Chaco* ecoregion (Córdoba, Argentina): An osteological approach to the characterization of a relict wild population

**DOI:** 10.1371/journal.pone.0194727

**Published:** 2018-04-11

**Authors:** Thiago Costa, Fernando Barri

**Affiliations:** 1 IDACOR, Museo de Antropología, Facultad de Filosofía y Humanidades, CONICET, Universidad Nacional de Córdoba, Córdoba, Argentina; 2 Instituto de Diversidad y Ecología Animal, CONICET—Universidad Nacional de Córdoba, Córdoba, Argentina; Illinois State Museum, UNITED STATES

## Abstract

Guanacos (*Lama guanicoe*) are large ungulates that have been valued by human populations in South America since the Late Pleistocene. Even though they were very abundant until the end of the 19th century (before the high deforestation rate of the last decades), guanacos have nearly disappeared in the *Gran Chaco* ecoregion, with relicts and isolated populations surviving in some areas, such as the shrubland area near the saline depressions of Córdoba province, Argentina. In this report, we present the first data from a locally endangered guanaco wild population, through the study of skeletal remains recovered in *La Providencia* ranch. Our results showed that most of the elements belonged to adults aged between 36 and 96 months; sex evaluation showed similar numbers of males and females. Statistical analysis of the body size of modern samples from Córdoba demonstrated that guanacos from the *Chaco* had large dimensions and presented lower size variability than the modern and archaeological specimens in our database. Moreover, they exhibited dimensions similar to those of modern guanacos from Patagonia and San Juan, and to archaeological specimens from Ongamira and Cerro Colorado, although further genetic studies are needed to corroborate a possible phylogenetic relationship. Finally, we used archaeozoological techniques to provide a first characterization of a relict guanaco population from the *Chaco* ecoregion, demonstrating its value to the study of modern skeletal remains and species conservation biology.

## Introduction

According to Raedeke’s [1] estimations, about 50 million guanacos (*Lama guanicoe*) would have lived in South America during pre-Hispanic times. Archaeological data from Argentina showed that these ungulates have been valued economically and ideologically by human populations in the Late Pleistocene and throughout the Holocene [2–11]. In the central mountains of Córdoba, an area of the *Gran Chaco* ecoregion, guanacos also would have played an important role in the lives of indigenous people [12–16].

Since the Spanish colonization, the *Gran Chaco* has suffered a transformation from natural areas to agricultural landscapes. However, in the last decades, due to technological and socio-economic advances, the habitat destruction increased exponentially, with millions of lost hectares of forests and savannahs. Currently, crops are produced (mainly soybean) along with cattle raising, causing great loss of biodiversity [17–20]. At the beginning of the 20th century, guanaco populations surviving in Córdoba mountain ranges were valued for their meat and skin; they also were hunted viciously for sport shooting ([21] p.340-342). Today, the few remaining guanacos are isolated in the shrublands near the saline depressions [22].

The era of anthropocene defaunation [23] has seen massive biodiversity extinction during the last century. Current wildlife populations are in marked decline [24]. New data from locally endangered wildlife populations are important for the conservation of species. The study of guanacos, large ungulates that can play an important role in the control of vegetation composition and structure [25–27], should include analysis of modern bone collections in order to obtain data for species protection. We used archaeozoological methods to offer new insights into changes that occurred in guanaco body sizes throughout the Holocene and in the modern population death profile. The techniques used for this study could advance the understanding of (a) the role that humans played in ecosystem changes, especially hunting and overexploitation during the early twentieth century ([21] p.339-345), and (b) the reasons for current declines of guanaco populations in Córdoba province.

Regional archaeological models have suggested that the numbers of guanacos in Córdoba could have been reduced since pre-Hispanic times due to human overexploitation [12]. Nonetheless, current guanacos are endangered in the *Chaco* area due to habitat loss associated with the introduction of European cattle, historical (colonial and capitalist) overexploitation, and urban expansion processes, among other anthropogenic factors associated with defaunation.

Paleozoological assemblages provide important data concerning anthropogenic and ecological processes that can be used in conservation biology [28–32]. In this report, we characterize age and sex of guanacos in modern bone remains from a relict population recovered from 7 sites in an area located in the *Corredor Biogeográfico del Chaco Árido*. For this purpose, we used common archaeozoological practices. We also used osteometric techniques to compare the body size of the modern sample with specimens from surrounding areas and local archaeological sites, to obtain a better definition of the variability in species size. Based on this comparison, we discuss size changes that may be associated with the isolation of the current population, as well as relationships with archaeological specimens.

Finally, this report aims to demonstrate that the analysis of a modern collection using archaeozoological techniques can offer new insights that should help develop a better conservation agenda for the locally endangered guanacos.

### Guanacos and humans, zooarchaeological data

Regional zooarchaeological data indicate that indigenous people used guanacos as a resource since the Early Holocene [12–16]. Nonetheless, Rivero *et al*. [12] argue that the use of wild camelid populations by humans, as their main resource, could have not been sustained beyond the Late Holocene (3000 B.P.). These interpretations are based on the zooarchaeological analysis of 14 sites dating to Early-Late Holocene and the apparent decreasing presence of *Lama* sp. remains over time [12].

Rivero & Medina [33] analyzed the archaeofaunal assemblages of 8 sites and introduced the records from 2 other assemblages (Quebrada del Real 1-Component 1, Quebrada del Real -Component 2) [12]. The authors suggest two hypotheses to explain reduced camelid density and increased numbers of smaller-sized animals in the zooarchaeological record. The first hypothesis suggests that participation of women and children in the economy contributed to hunting or collection of the smaller animals. The second hypothesis proposes that the growth in the human population, with resulting overexploitation of camelids, was the main factor that led humans to seek other resources [33].

Mitochondrial DNA analysis of past humans in the area suggests that, by *ca*. 1200 B.P., significant changes had occurred in the population from the high plains. Those changes included an increment in haplogroup B that modified the previously-dominant gene pool in the area (haplogroup C) [34]. Moreover, different proxies for paleoclimate indicate an increase in humidity that started in the Middle Holocene, evidenced by a continuous replacement of C_4_ with C_3_ plants since *ca*. 3870 B.P. [35, 36]. These changes from open (grasses, C_4_) to denser vegetation (herbaceous or woody C_3_ species), probably affected faunal distribution because species had to adapt to these changing climate conditions. Accordingly, the causes of the decrease of guanacos in the archaeological record should be interpreted cautiously, since changes in both climate and human populations took place during the Late Holocene. Finally, in agreement with ([33] p. 84), “sampling bias and chronological gaps” should be eliminated by developing “regional scale studies”.

**Fig 1:**
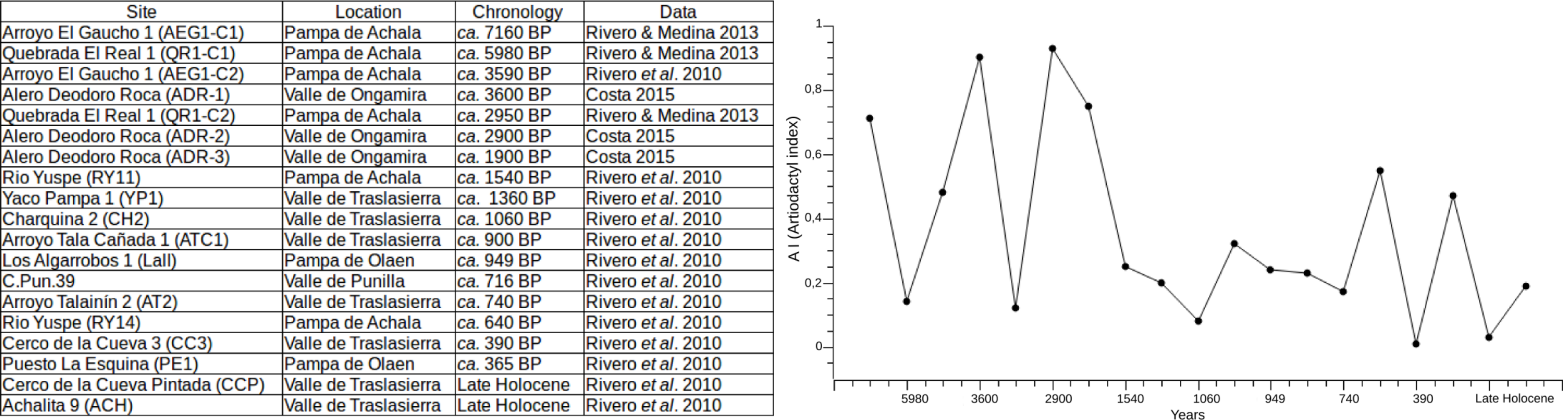
Artiodactyl index in archaeological sites throughout the Holocene. Late Holocene sites have relative dating *ca*. 1000–300 B.P. [12].

Fig 1 shows data taken from Rivero *et al*. [12] and Rivero & Medina [33] with the addition of samples from Alero Deodoro Roca site in Ongamira [15]. Artiodactyl index (∑ Artiodactyl / (∑ Artiodactyls + ∑ small vertebrate) suggests a decrease in the human use of large ungulates (*Lama* sp. represents 77.49% of the ungulate number of identified species -NISP) throughout the Holocene.

## Material and methods

**Fig 2:**
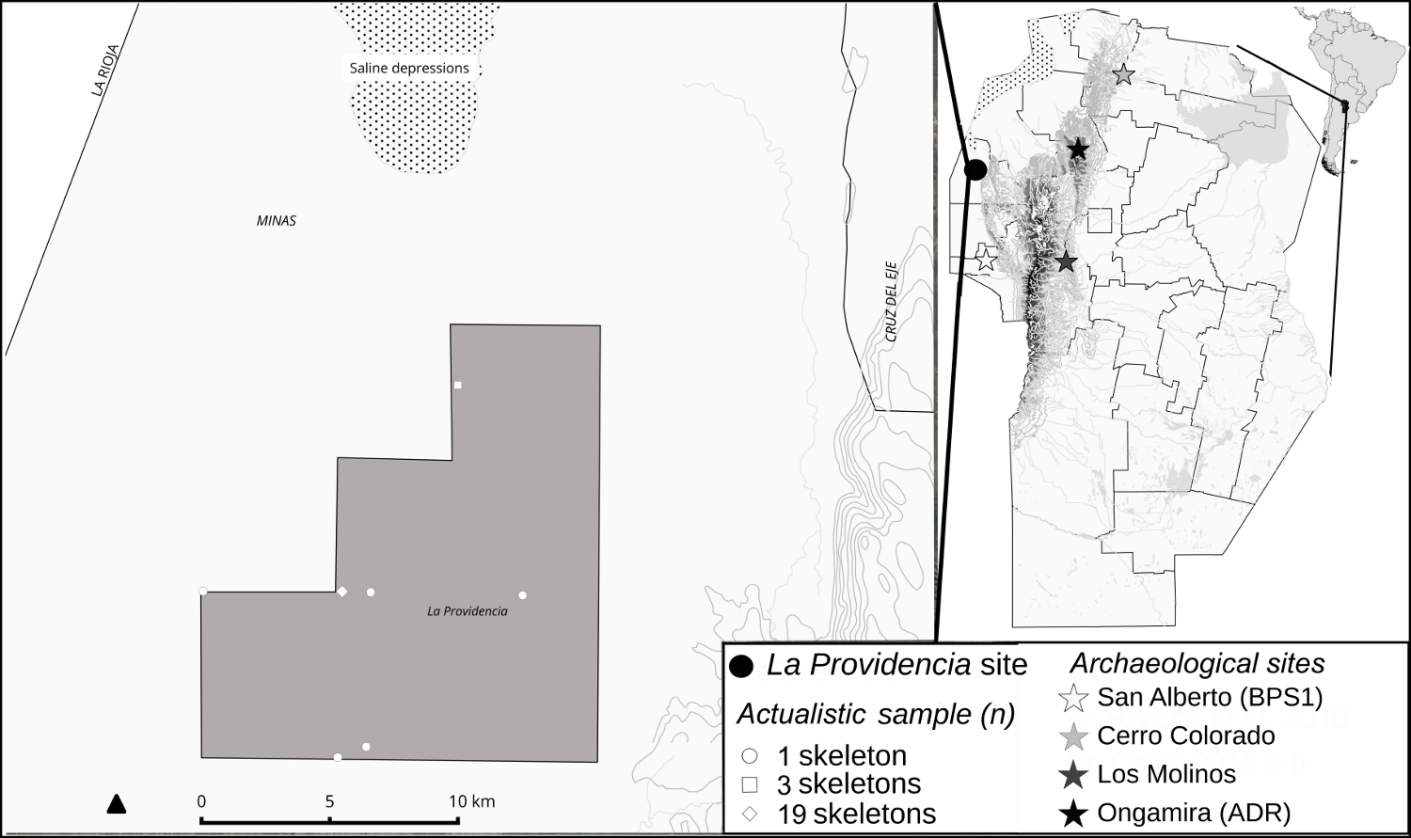
Study area. Upper right: map showing Córdoba province, its location in South America and the locations of the modern and archaeological sites discussed in this report. Left: Detail of the collection points in *La Providencia* ranch (30°56'54.44"S, 65°34'34.93"W).

During the second semester of 2015, we surveyed the 200,000 hectares of the *Salinas Grande* area, searching for guanacos remaining in Córdoba. We found one population on *La Providencia* ranch, where we recovered 413 complete bone elements belonging to a minimum of 27 skeletons (Fig 2). Nineteen specimens had been killed and discarded in the same area through illegal poaching. The remaining 8 were dispersed on the ranch, and from this sample, 5 individuals died caught in the wires delimiting the field (2 females, 1 male, and 2 specimens of unidentified sex). We were not able to determine the cause of death of the remaining 3 guanacos.

Weathering conditions of the samples agree with those reported previously for juvenile and adult guanaco bones exposed to subaerial deterioration for periods no longer than 48 months [37–39]. S1 Table displays the maximum weathering stages [40] and their description for the 50 bone elements analyzed in this report. Cranial elements exhibited more advanced weathering stages of frontal and supraorbital bones, compared to maxillary bones. The measured phalanges and alveolar processes from maxilla and mandible bones showed no signs of weathering.

### Age estimation

Age estimation through dental development, eruption, and wear is a common ageing method in the archaeozoology of camelids and in the biology of guanacos [1, 4, 41–45]. Guanacos have heterodont and diphyodont dentition, with 4 types of teeth: incisors (I), canines (C), premolars (P), and molars (M) [1, 43, 44].

We used Kaufmann’s [4] sequence of tooth development and wear for upper and lower guanaco teeth to characterize the age of 30 cranial and mandibular remains. This classification was based on observation of tooth (P, M, and deciduous premolars Pd) development and wear from Rio Negro, Argentina. The following categories were defined: unborn (10–11 months of gestation), offspring (0–12 months), juvenile (12–24 months), subadult (24–36 months), adult (3–10 years), and senile (over 10 years) [4].

### Sex evaluation

It is accepted widely that South American camelids do not have sexual dimorphism [1, 4, 41]. Nonetheless, size of the canine teeth is one characteristic that can be determinant in assigning sex within species where only males must compete for territories [1, 43]. Raedeke ([1] p. 99) states that, to determine sex in guanacos, considering the overall mass of the canines yields better results than considering their length. The author proposes a “canine index”, which is calculated by multiplying the height of the canine by its maximum diameter at the alveolar rim [1].

Following Raedeke’s [1] suggestion, Lefèvre *et al*. [46] adapted the procedure to samples in which canines were not present. These authors took 2 measurements of the mandibular canine alveolar process, the maximum and minimum diameters ([46] p. 79). Using this technique, they were able to discriminate a group of males with a maximum diameter above 10 mm and a female group having this diameter below 9 mm [46]. To determine the sex of individuals in our samples, we followed the proposal of Lefèvre *et al*. [46] for the specimens categorized as adults and, whenever possible, compared these results with the different characteristics of the male/female pelvis described by Kauffman [4, 47]. We calculated the geometric mean of the variables measured in the same units (mm), a well- discussed and commonly-used morphological proxy for skeletal size measurements that can be used to understand geometric similarity [3, 16]. Results are presented using bivariate graphs and mixture analysis [47, 48].

### Body size

Determining morphotypes in artiodactyl populations allows us understand how they are adapted to their environment [49]. For example, larger body size directly affects relationships with other species, influencing ability to compete for resources or to escape from predators [50]. To characterize body size, we compared data from 5 osteometric variables: 1) maximum length of phalanges, taken parallel to the major axis based on the tangent formed by the proximal and end plantar condyles at the most distal point of the distal articular surface; 2) width of the proximal articular surface; 3) height of the proximal articular surface; 4): width of the distal articular surface; 5) height of the distal articular surface [41, 51]. This procedure was done with 10 first modern phalanges from Córdoba, having the same variables as those of 14 samples recovered from 4 regions of Argentina (Salta, Catamarca, San Juan, and Patagonia) [52]. To determine differences over time, we added 10 phalanges from local archaeological sites (Fig 2) for comparison. We used only forelimb phalanges, since they were found to better discriminate the differences in body size of the specimens than hind limb phalanges [52]. We performed multivariate cluster analysis and mixture analysis to characterize size differences among the study specimens [51–53]. For the statistical analysis we used the software Past version 3.14 [54].

## Study area

The study area was located on the *La Providencia* ranch (Fig 2), an area situated in the *Corredor Biogeográfico del Chaco Árido* near the *El Chacho* locality (Córdoba, Argentina). According to Zak and Cabido [55], the area belongs to the lowland region, classified as *Aspidosperma quebracho-blanco* and *Prosopis nigra* forest, which is being displaced rapidly by cultural landscapes and shrublands. The soils are characterized by extreme salinity, with noticeable accumulation of carbonates or gypsum and saline layers [56].

The climate in the region is mild, according to the characteristics of a semiarid region. Temperature mean is about 25°C during summer and approximately 12°C in winter. Precipitation ranges between 130 mm in summer and is below 40 mm during the coldest months (data from the Argentina’s meteorological service for Villa de María de Río Seco, available at: http://www.smn.gov.ar/serviciosclimaticos/). Romero & Santa María [56] presented rainfall data recorded at *La Providencia*. According to the authors, precipitation was 165 mm between February and December 2013, 536 mm between January and December 2014, 607 mm between January and December 2015, and 349 mm from January to September 2016 ([56] p. 18).

## Results

### The modern sample

Age estimates showed that most of the elements recovered (n = 20) belonged to adults aged from 36 to 96 months (Table 1). The classification used here is better suited to the mandibular sequence of tooth development and wear than to maxillary tooth sequence. This was evident in the comparison of the results of maxilla (CRNMF3) and mandibular (MRMMF2) bones belonging to a guanaco that died trapped in the fences of *Molino Ferrarini* (MF). The mandibular bone MRMMF2 exhibited better resolution of the estimated age of death (5 to 6 years) than the cranial bone (CRNMF3). Furthermore, 15% of the elements belonged to young adults (A1), 55% of the elements were guanacos that died at between 48 and 84 months of age (A2), 10% of the recovered bones aged from 5 to 6 years (A3). The remaining 20% were aged between 6 and 8 years (A4 and A5).

**Table 1:** Age estimation for 30 cranial (MX) and mandibular (MR) guanaco bones. Age group according to Kauffman’s [4] categorization (J = juvenile; S = subadult; A = adult).

Code	Element	Tooth wear (*sensu* Kauffman 2009)	Age group	Age (months)
MRMF1	MR	Pd4 exhibits moderate wear, M1 is emerging and M2 is in the alveolus	J1	12 to 19
MRCF16	MR	Pd4 is highly worn, M1 has minimum wear, M2 is almost fully developed and M3 is in the alveolus	S1	24 to 30
MRCF15	MR	P3 is absent, P4 shows wear, M1 is highly weared, M2 wear is moderate and M3 has some wear	A2	48 to 60
MRCF24	MR	Pd3 is absent, M1 is highly worn, M2 and M3 wear is moderate	A3	60 to 72
MRMF2	MR	Pd3 is absent, M1 is highly worn, M2 and M3 wear is moderate	A3	60 to 72
MRCF25	MR	M1 is highly worn and without infundibulum, M2 has moderate wear, M3 has moderate wear	A4	72 to 84
MRCF23	MR	M1 is highly worn without infundibulum, M2 has moderate to high wear, M3 has moderate wear	A5	84 to 96
CRNMF1	MX	Pd3 and Pd4 exhibit moderate wear, M1 is emerging and M2 is in the alveolus	J1	12 to 19
CRNMF2	MX	Pd3 and Pd4 exhibit moderate wear, M1 is emerging and M2 is in the alveolus	J1	12 to 19
CRNCF12	MX	Pd2 is lost, Pd3 and Pd4 are highly worn, M1 shows wear, M2 has almost no wear, M3 is in the alveolus	S2	30 to 36
CRNCF14	MX	Pd2 is lost, Pd3 and Pd4 are highly worn, M1 shows wear, M2 has almost no wear, M3 is in the alveolus	S2	30 to 36
CRNCF19	MX	Pd2 is lost, Pd3 and Pd4 are highly worn, M1 shows wear, M2 has almost no wear, M3 is in the alveolus	S2	30 to 36
CRNCF5	MX	Pd2 is lost, Pd3 and Pd4 are highly worn M1 shows wear, M2 has almost no wear, M3 is in the alveolus	S2	30 to 36
CRNCF7	MX	Pd2 is lost, Pd3 and Pd4 are highly weared, M1 shows wear, M2 has almost no wear, M3 is in the alveolus	S2	30 to 36
CRNNO22	MX	Pd2 is lost, Pd3 and Pd4 are highly worn, M1 shows wear, M2 has almost no wear, M3 is in the alveolus	S2	30 to 36
CRNCF17	MX	P4 is active and may show dentine, M1 has moderate wear, M2 is active and may show some wear, M3 erupts	A1	36 to 48
CRNCF18	MX	P4 is active and may show dentine, M1 has moderate wear, M2 is active and may show some wear, M3 erupts	A1	36 to 48
CRNCF3	MX	P4 is active and may shows dentine, M1 has moderate wear, M2 is active and may show some wear, M3 erupts	A1	36 to 48
CRNCF1	MX	P4 shows moderate wear, M1 is highly worn, M2 wear is moderate to high and M3 has moderate wear	A2	48 to 84
CRNCF10	MX	P4 shows moderate wear, M1 is highly worn, M2 wear is moderate to high and M3 has moderate wear	A2	48 to 84
CRNCF11	MX	P4 shows moderate wear, M1 is highly worn, M2 wear is moderate to high and M3 has moderate wear	A2	48 to 84
CRNCF13	MX	P4 shows moderate wear, M1 is highly worn, M2 wear is moderate to high and M3 has moderate wear	A2	48 to 84
CRNCF2	MX	P4 shows moderate wear, M1 is highly worn, M2 wear is moderate to high and M3 has moderate wear	A2	48 to 84
CRNCF20	MX	P4 shows moderate wear, M1 is highly worn, M2 wear is moderate to high and M3 has moderate wear	A2	48 to 84
CRNCF21	MX	P4 shows moderate wear, M1 is highly worn, M2 wear is moderate to high and M3 has moderate wear	A2	48 to 84
CRNCF6	MX	P4 shows moderate wear, M1 is highly worn, M2 wear is moderate to high and M3 has moderate wear	A2	48 to 84
CRNCF8	MX	P4 shows moderate wear, M1 is highly worn, M2 wear is moderate to high and M3 has moderate wear	A2	48 to 84
CRNMF3	MX	P4 shows moderate wear, M1 is highly worn, M2 wear is moderate to high and M3 has moderate wear	A2	48 to 84
CRNCF4	MX	P3 is absent in most cases, P4 shows moderate wear, M1, M2 and M3 are highly worn	A5	84 to 96
CRNCF9	MX	P3 is absent in most cases, P4 shows moderate wear, M1, M2 and M3 are highly worn	A5	84 to 96

Table 1 also indicates the results from 10 elements categorized as subadult guanaco. Within this group, 3 elements (2 individuals) were aged between 12 and 19 months (J1). The estimated age at death of the remaining 7 elements was between 24 and 36 months. It should be noted that the only mandibular (MRCF16) element in the S age group showed an estimated age of 24–30 months (S1). Therefore, we considered that the element could belong to one of the cranial remains categorized as S2, even though we were not able to rejoin the cranial-mandibular pieces due to a trampling fracture of the coronoid process of the mandibular section.

**Fig 3:**
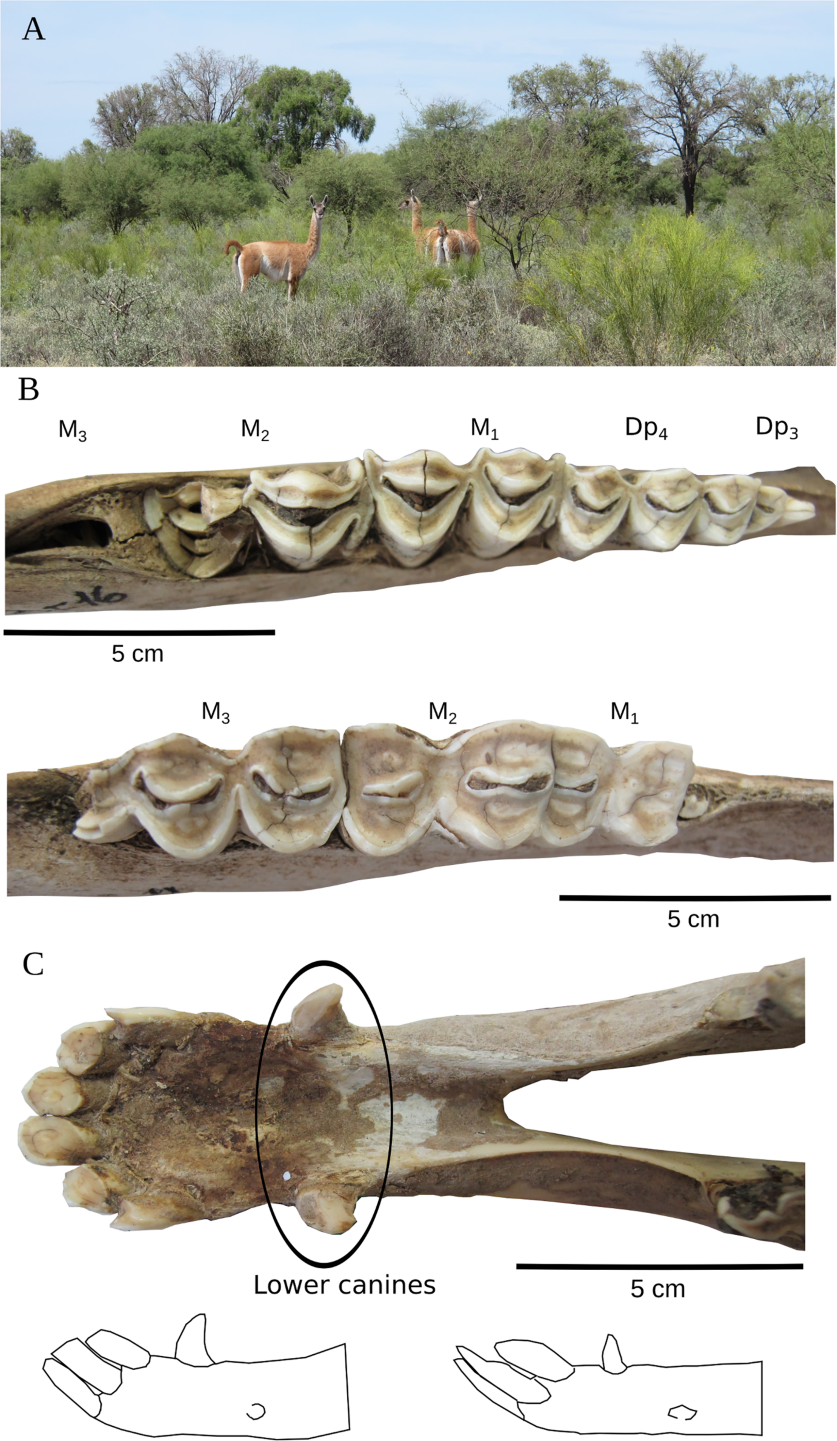
Lama guanicoe. A: A group of reproductive guanacos. Fig 3B: (above) Mandibular teeth (S1 age group) reveals deciduous premolars (Dp) and molars (M); (below) mandibular molars of an adult guanaco (A3 age group). Fig 3C: (above) lower canines of a male specimen (MRMF2, A3 age group); (below) example of the differences in the lower canines; male on the left side of the scheme and female guanaco on the right (see also Kaufmann [4]).

Fig 3 displays a group of guanacos on *La Providencia* ranch (A). The mandibular teeth of a subadult (MRCF16) guanaco include deciduous premolars 3 and 4 (D_p_) and the alveolar process with M_3_ inside. Likewise, the mandible seen in Fig 3B shows the 3 molars of an adult guanaco with an estimated age at death between 5 and 6 years (MRCF24). Comparing these two mandibles revealed the differences in molar wear. The infundibulum, the funnel-shaped cavity, is nearly lost in the adult’s M1. Moreover, the adult mandible lacks the deciduous premolars and M_3_ already is modified by attrition; the enamels clearly are separated from the dentin (Fig 3B below).

Fig 3 also reveals qualitative information that contributes to sex discrimination (n = 19 elements); male canines are more robust and curved than female ones (C). Nonetheless, in osteological collections recovered after a few years of weathering exposure, some teeth tend to fall from the alveolar process (in our study, most of these were canines). Hence, whenever possible, the whole specimen (and/or other attributes such as pelvis) should be compared in any research.

**Fig 4:**
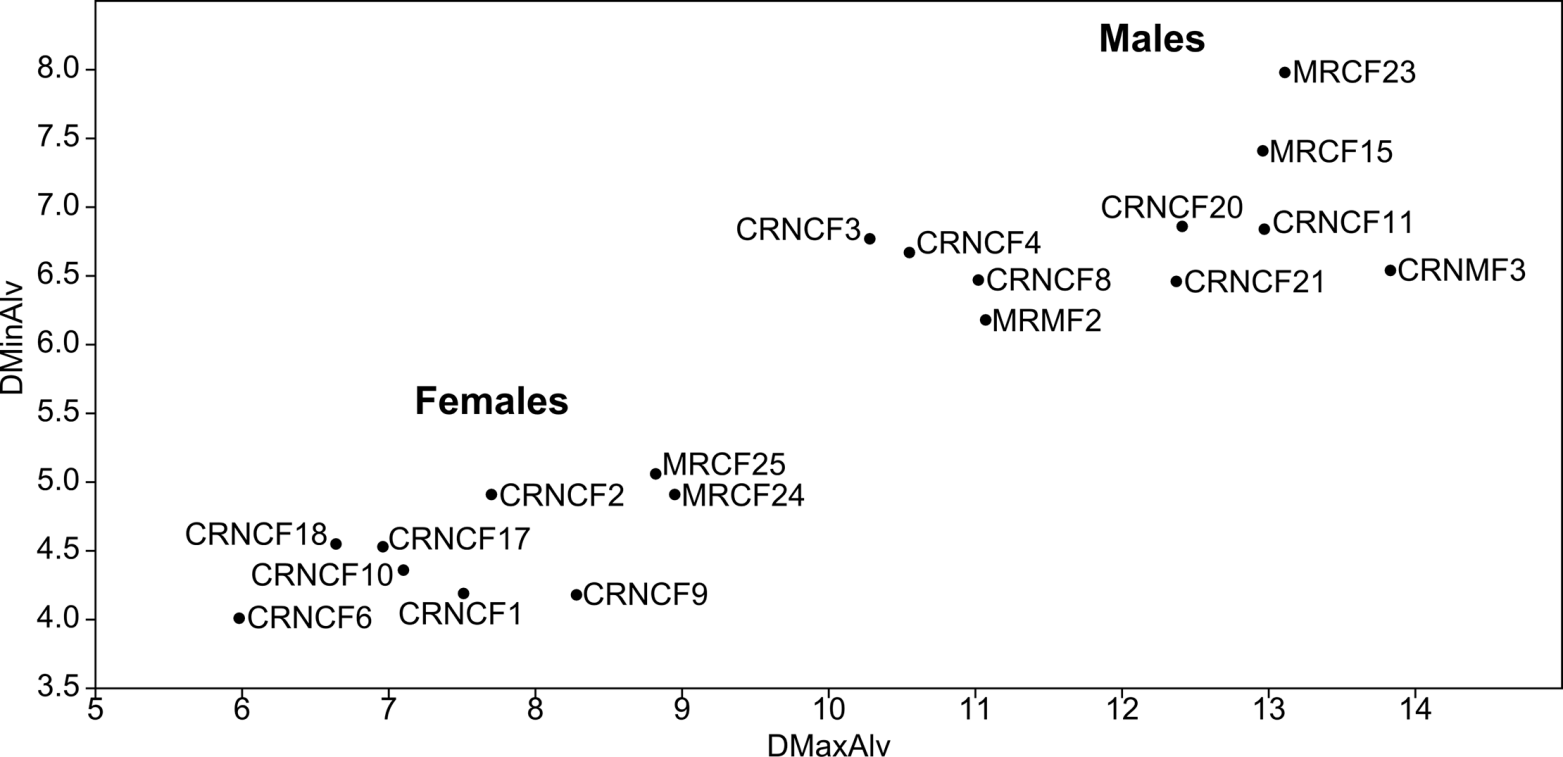
Bivariate graph. Results of sex categorization according to the diameter of the alveolar process.

The results obtained from the measurement of the alveolar process revealed a clear differentiation of the specimens into two groups. One group has larger dimensions (9.44 mm mean in both measurements) and is composed of 10 specimens identified as males (Fig 4). The other group has a mean of 6.04 mm, and is composed of 9 specimens that, following Lefèvre *et al*. [46], correspond to the measurement of females (Fig 4). Our results are similar to those reported previously, supporting the use of this technique when differentiating sex groups in a guanaco osteological collection. In addition, we compared our results with those described by other authors [4, 47] regarding the characteristics of the pelvis in specimen CRNMF3, and found the same results.

We performed a mixture analysis of the geometric mean of both measurements described in Materials and Methods to corroborate the bivariate results and found the same distribution (Table 2). Therefore, we suggest that the technique presented by Lefèvre *et al*. [46] is accurate for separating males and females.

**Table 2:** Results of the mixture analysis. Geometric mean of maximum and minimum diameters of the canine alveolar process.

Assignments	Group 1	Group 2	Max group
CRNCF1	0.00	**0.32**	2
CRNCF2	0.00	**0.30**	2
CRNCF3	**0.18**	0.01	1
CRNCF4	**0.19**	0.00	1
CRNCF6	0.00	**0.08**	2
CRNCF8	**0.21**	0.00	1
CRNCF9	0.00	**0.35**	2
CRNCF10	0.00	**0.31**	2
CRNCF11	**0.27**	0.00	1
MRCF15	**0.17**	0.00	1
CRNCF17	0.00	**0.32**	2
CRNCF18	0.00	**0.29**	2
CRNCF20	**0.31**	0.00	1
CRNCF21	**0.32**	0.00	1
CRNMF3	**0.25**	0.00	1
MRMF2	**0.16**	0.01	1
MRCF23	**0.06**	0.00	1
MRCF24	0.00	**0.12**	2
MRCF25	0.00	**0.10**	2

### Characterizing variation in body size throughout the Holocene

According to results of body size (Table 3), the largest geometric mean (27.16 mm) corresponded to the modern specimens from Córdoba, with Patagonian specimens showing 25.72 mm and only one individual above 27 mm (MLP-G4). These results could reflect sampling, since Patagonian guanacos are expected to be larger (we are aware of body size differences among Patagonian specimens, but we have no precise data regarding site of origin of the latter). Northwestern specimens presented a geometric mean of 24.32, whereas the mean of specimens from San Juan was closer to that of Córdoba sample (26.29 mm).

**Table 3:** Measured phalanges. Measurements in millimeters (1–5) and geometric mean of 34 forelimb first phalanges (for radiocarbon dates from Ongamira samples, see [57]).

Time Period	Location	Code	1	2	3	4	5	Mean	Data source
Modern	Patagonia	MLP-G1	75.00	22.00	21.00	19.00	17.00	25.69	Izeta *et al*. 2009
Modern	Patagonia	MLP-G2	75.00	22.00	20.00	19.00	17.00	25.44	Izeta *et al*. 2009
Modern	Patagonia	MLP-G3	73.00	23.00	19.00	19.00	17.00	25.27	Izeta *et al*. 2009
Modern	Patagonia	MLP-G4	85.00	23.00	22.00	20.00	18.00	27.41	Izeta *et al*. 2009
Modern	Patagonia	MLP-G5	74.00	24.00	19.00	19.00	17.00	25.56	Izeta *et al*. 2009
Modern	Patagonia	MLP-G6	75.00	22.00	21.00	19.00	15.00	25.06	Izeta *et al*. 2009
Modern c	Catamarca	G149-4	72.80	21.50	18.10	17.80	16.70	24.27	Izeta *et al*. 2009
Modern	Catamarca	G149-5	71.90	21.50	18.40	18.70	16.30	24.41	Izeta *et al*. 2009
Modern	Catamarca	G149-6	71.70	22.20	18.50	18.70	16.70	24.70	Izeta *et al*. 2009
Modern	Catamarca	G149-7	72.80	21.80	18.40	18.20	16.90	24.59	Izeta *et al*. 2009
Modern	Salta	G1100-1	71.32	19.65	18.37	17.58	16.29	23.63	Izeta *et al*. 2009
Modern	San Juan	FPDG-1	78.36	22.65	21.44	19.07	17.72	26.41	Izeta *et al*. 2009
Modern	San Juan	FPDG-2	75.90	22.04	21.02	18.64	18.38	26.07	Izeta *et al*. 2009
Modern	San Juan	FPDG-3	77.72	22.67	20.53	19.20	18.37	26.37	Izeta *et al*. 2009
Modern	Córdoba	MF3	81.06	23.08	21.82	19.07	17.42	26.70	Costa & Barri in this paper
Modern	Córdoba	CF1	87.73	23.95	22.71	21.14	18.53	28.47	Costa & Barri in this paper
Modern	Córdoba	CF2	88.26	23.56	22.26	21.41	19.06	28.53	Costa & Barri in this paper
Modern	Córdoba	CF4	81.31	22.44	21.32	20.67	17.54	26.91	Costa & Barri in this paper
Modern	Córdoba	CF5	81.28	22.85	21.91	20.24	17.48	27.02	Costa & Barri in this paper
Modern	Córdoba	CF6	80.97	23.05	20.81	19.47	17.63	26.61	Costa & Barri in this paper
Modern	Córdoba	CF7	83.44	22.37	21.75	19.85	18.24	27.13	Costa & Barri in this paper
Modern	Córdoba	CF8	82.89	22.17	21.97	19.87	17.06	26.75	Costa & Barri in this paper
Modern	Córdoba	CF16	86.07	21.90	21.78	19.03	18.44	27.02	Costa & Barri in this paper
Modern	Córdoba	CF20	84.47	21.60	21.27	17.56	19.06	26.47	Costa & Barri in this paper
*ca*. 2900 B.P.	Ongamira	443	76.63	21.80	20.75	17.83	17.55	25.53	Costa & Barri in this paper
*ca*. 2900 B.P.	Ongamira	1357	76.50	21.36	19.78	17.71	14.22	24.11	Costa & Barri in this paper
*ca*. 3600 B.P.	Ongamira	1535	71.83	20.86	19.87	16.95	15.02	23.76	Costa & Barri in this paper
*ca*. 2900 B.P.	Ongamira	3810	79.18	23.77	20.71	18.60	18.27	26.57	Costa & Barri in this paper
*ca*. 2900 B.P.	Ongamira	3871	72.58	23.67	20.73	19.01	17.53	25.99	Costa & Barri in this paper
Late Holocene (4000–1500 B.P.)	Ongamira	60–172	76.61	21.49	21.77	19.68	19.66	26.82	Costa & Barri in this paper
Late Holocene (*ca*. 1000 B.P.)	San Alberto	BPS191	75.52	19.76	20.04	17.56	16.08	24.28	Costa & Barri in this paper
Late Holocene (*ca*. 1000 B.P.)	San Alberto	BPS192	75.41	19.31	19.82	17.52	16.59	24.25	Costa & Barri in this paper
Late Holocene (*ca*. 1000 B.P.)	Los Molinos	62/2-1	69.29	18.93	18.21	16.54	14.60	22.50	Costa & Barri in this paper
Late Holocene (*ca*. 1000 B.P.)	Cerro Colorado	AJPHF2	76.45	22.61	22.15	19.36	16.90	26.28	Costa & Barri in this paper

The 10 archaeological modern samples from Córdoba had a geometric mean of 25.15 mm. Samples from Ongamira had a mean of 25.46 mm, with the element dated to 3600 B.P. measuring 23.76 mm, the lowest in the group. Elements 3810 (26.57 mm) and 60–172 (26.82 mm) from Ongamira had the closest geometric means compared to the mean of our modern samples.

The elements from San Alberto revealed the smallest (24.28 and 24.25 mm) means and presented similar dimensions, suggesting that the phalanges might belong to the same individual. The element found at Los Molinos had the smallest geometric mean (22.50 mm) in the database; the element found at Cerro Colorado had a mean of 26.28, closer to the mean of the phalanges from Ongamira, San Juan, and Córdoba than that of Los Molinos (Table 3).

**Fig 5:**
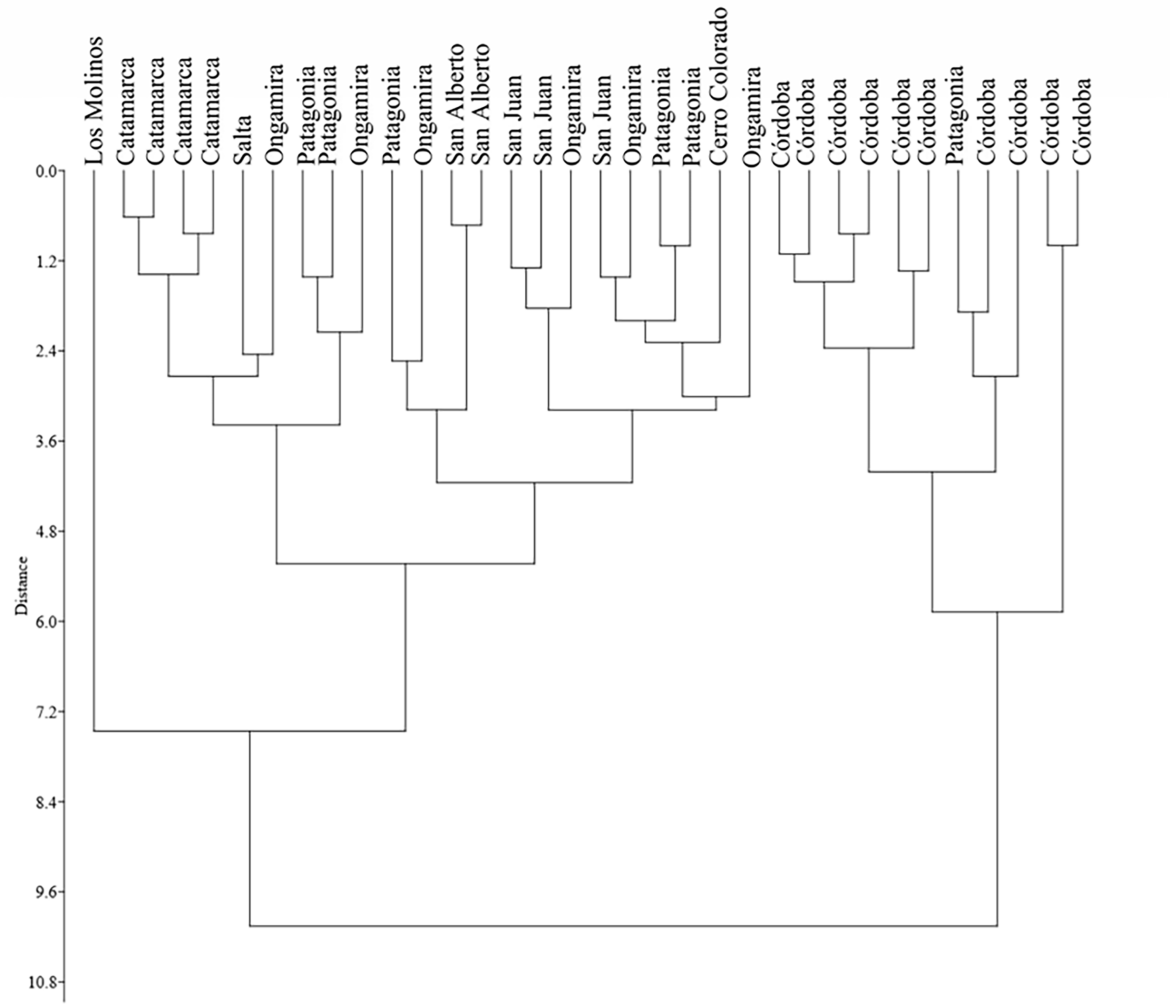
Cluster analysis (UPGMA). UPGMA showing body size differences in Argentine guanacos.

Multivariate paired group (UPGMA) with Euclidean similarity index was used to create a cluster that separates the samples. Fig 5 shows 3 groups differing in body size. The small size group includes only one archaeological specimen from Los Molinos. The medium size assemblage includes 64% of the specimens we analyzed and can be divided into 2 subgroups. One of these gathers a smaller size class, and includes the modern samples from Catamarca, Salta, specifically 2 phalanges from the Patagonian sample (MLP-G3 and MLP-G5), and 2 phalanges from Ongamira (1535 and 3871). The other subgroup includes specimens from Patagonia (MLP-G1, MLP-G2, and MLP-G6), Ongamira (1357, 3810, 443, and 60–172), San Alberto (BPS-191 and BPS-192), San Juan (FPDG-1, FPDG-2, and FPDG-3), and the element from Cerro Colorado (AJPHF2). Finally, the large size group consists of modern phalanges from Córdoba (CF and MF3) and one recovered in Patagonia (MLP-G4).

Table 4 shows Mixture Analysis results using the geometric mean of the 5 measured variables. The maximum-likelihood method allowed us to segregate the samples into 2 groups. One group included 23 phalanges with larger dimensions: 18 modern samples including Patagonia (n = 5); all samples from San Juan and Córdoba; and 5 archaeological elements from Ongamira and Cerro Colorado. The other group included 11 elements with smaller dimensions: 1 phalange from the modern samples from Patagonian (MLP-G6) and all the phalanges from northwestern Argentina (Catamarca and Salta); and 2 phalanges from Ongamira (1357 and 1535), San Alberto, and Los Molinos.

**Table 4:** Results of the mixture analysis. Geometric mean of the 5 variables measured from the first phalanges.

Assignments	Group 1	Group 2	Max group
**Patagonia**	**0.190**	0.034	1
**Patagonia**	**0.141**	0.057	1
**Patagonia**	**0.110**	0.077	1
**Patagonia**	**0.184**	0.000	1
**Patagonia**	**0.165**	0.045	1
Patagonia	0.078	**0.103**	2
Catamarca	0.013	**0.168**	2
Catamarca	0.018	**0.166**	2
Catamarca	0.037	**0.146**	2
Catamarca	0.029	**0.155**	2
Salta	0.002	**0.120**	2
**San Juan**	**0.292**	0.005	1
**San Juan**	**0.258**	0.013	1
**San Juan**	**0.290**	0.005	1
**Córdoba**	**0.290**	0.002	1
**Córdoba**	**0.029**	0.000	1
**Córdoba**	**0.025**	0.000	1
**Córdoba**	**0.270**	0.001	1
**Córdoba**	**0.255**	0.000	1
**Córdoba**	**0.294**	0.002	1
**Córdoba**	**0.237**	0.000	1
**Córdoba**	**0.287**	0.001	1
**Córdoba**	**0.255**	0.000	1
**Córdoba**	**0.294**	0.004	1
**Ongamira**	**0.159**	0.048	1
Ongamira	0.008	**0.164**	2
Ongamira	0.003	**0.136**	2
**Ongamira**	**0.295**	0.003	1
**Ongamira**	**0.246**	0.016	1
**Ongamira**	**0.281**	0.001	1
San Alberto	0.013	**0.168**	2
San Alberto	0.012	**0.168**	2
Los Molinos	0.013	**0.014**	2
**Cerro Colorado**	**0.284**	0.007	1

## Discussion

The guanaco has nearly disappeared in the *Gran Chaco*, with only two or three relict populations currently surviving in the opposite extremes of the eco-region distribution: one or two in the north, between Bolivia and Paraguay [58], and the other in the south, as described in this report. The decline of the *Chaco* guanaco population results from multiple factors affecting wildlife in the region, such as habitat loss, interspecific competition with livestock, and hunting [59, 60].

Bioarchaeological studies indicate that *ca*. 1200 B.P, there was a process of human migration and change in way of life [36, 61]. This process might have been associated with environmental pressures, such as climate change, demographic increases in population sizes, and development of new social organizations, probably with the emergence of a more hierarchical structure [61]. Regional archaeofaunal data suggest that despite the environmental and population changes, guanaco hunting remained an important economic activity [13–16].

Regarding the reasons for camelid decline in the archaeofaunal record of *Gran Chaco* province during pre-Hispanic times (as defined by the artiodactyl index [33]), 15 zooarchaeological assemblages should be interpreted cautiously. Even though not all of the 2017 recorded sites in the province [62] present comparable archaeofaunal samples, further research is needed to avoid sampling bias. Moreover, historical and ethnohistorical data also indicate that guanaco hunting persisted in the mountain chains until the beginning of the 20th century [21, 63], and still occurs in the southern *Salinas Grandes*.

Results from sex estimation in the extant population from Córdoba presented a similar number of adult males and females, with the number of adults being twice that of the subadults. However, most of our sample (n = 19) comes from illegal poaching activities that probably occurred between 3–4 years ago, according to information gathered from local dwellers and supported by the weathering stages of the specimens (S1 Table). Moreover, the mortality profile of the sample recovered from poaching activities shows that 6 elements belonged to subadults (24–36 months old), 13 elements belonged to adults (3–6 years old) and 5 elements belonged to older adults. Thus, the addition of individuals of different age groups (with a higher number of 3–6 years old adults) and of both sexes to the poaching sample suggests a hunting strategy that included guanaco family groups and young male groups, as discussed by other authors ([4] p. 389). In addition, the individuals that died caught in the fences tended to be younger (J1 and S2 age groups), as expected due to their lack of experience [4].

Guanaco mating is based on polygyny, for protection of the population. Puberty is reached around age 2 years in females and 3 years in males. However, due to the high competition in the wild, males under age 5 seldom reproduce under. Therefore, our study sample might contain more reproductively active females than males, since our estimated age for males included at least 5 (considering MX only) individuals that could be under reproductive age [64]. It would be necessary to collect more specimens with more complete skeletons to estimate sex differences using other identifying metrics (such as pelvis shape). This would allow better characterizing of subadults, since the osteometric approach cannot be applied when bone development is incomplete. On the other hand, results from mixture analysis revealed the same distribution of sex estimation as that observed in the bivariate graph, suggesting that the technique can be an important tool for differentiating sex of fragmented skeletons.

Our results from body size analysis demonstrate that modern specimens from Córdoba have larger dimensions than those of modern and local archaeological specimens from other regions. Moreover, mixture analysis grouped the modern sample with specimens from Patagonia and San Juan, the specimens of largest dimensions in our database. Hence, we propose that the differences in body size might be related to some adaptation to the *Chaco* native forests in Córdoba. In addition, the archaeological samples from Córdoba showed higher variability in their dimensions than the modern sample from *Chaco*.

Late Holocene (*ca*. 1000 B.P) archaeological specimens from San Alberto and Los Molinos revealed smaller dimensions (geometric mean of 23.68 mm) than the one from Cerro Colorado (geometric mean of 26.28 mm, see Table 3). Phalanges from Ongamira mostly showed geometric means above 25 mm, except for two specimens dated to 2900 B.P. (1357) and 3600 B.P. (1535). A previous work in the regional characterization of archaeological camelids presented similar results. Despite the size variability, most specimens tended to be medium or large size [13, 16]. Finally, mitochondrial DNA data suggest the existence of a guanaco metapopulation that replaced the extinct Patagonian guanacos at *ca*. 10551 years ago, becoming ancestral to all modern guanacos [65]. Thus, ancient guanacos from Córdoba probably bred with different populations, which may have increased size variability.

Even though there would be only two subspecies of guanacos, the Peruvian *L*. *guanicoe cacsilensis* and the remaining populations grouped in the clade recognized as *L*. *g*. *guanicoe* [66], our data suggest that guanacos from *Chaco* might represent a particular ecotype. This hypothesis must be tested with further anatomical, demographic, and genetic studies. Therefore, considering also that mammals play a particularly important role in the dynamics of the *Chaco* ecosystem, we propose that a species conservation status for these unique guanaco population should be established.

Additionally, it would be important to compare our data with those of other persisting *Chaco* guanaco populations from Bolivia and Paraguay [58]. We should enlarge our database by adding samples from other archaeological or paleontological time periods to obtain better resolution for body size variation of guanacos in the *Chaco* region.

## Final comments

Our results demonstrate that the modern sample from Córdoba has the largest dimensions of the phalanges represented in our database. Nevertheless, our data should be compared with those from archaeofauna and paleontological guanaco remains in the area. Furthermore, new modern samples (especially from *Chaco* areas where the species is still present) should be added to the analysis if we are to fully understand the requirements for the conservation of the extant population.

The guanacos are important because they are the last remnant population in an area where guanacos historically were numerous [1]. They now are in decline due to anthropogenic environmental and climate changes, particularly during recent decades, wherein habitat loss in the *Chaco* ecoregion has increased dramatically [19,20]. Therefore, it is essential to understand how colonial development with the introduction of European cattle, vegetation changes, and commercialization of native animal skins, could have affected guanacos in Córdoba ([21] p. 349, [66]). Moreover, historical data for Argentina indicate that native products incorporated to the capitalist system brought several faunal populations to the brink of extinction [67,68, 69].

Finally, although the archaeofaunal assemblages in Córdoba remain fragmentary, sampling and chronological bias tends to diminish as research advances. Improved temporal resolution from Holocene paleozoological collections should provide new insights into conservation biology [29]. Accordingly, as Barnosky *et al*. [32] suggest, fostering conservation planning and practice requires the use of paleozoological and historical information.

## Supporting information

S1 Table Maximum weathering stages of the analyzed sample. (DOCX)
