# Supplementary material for: Lama guanicoe remains from the Chaco ecoregion (Córdoba, Argentina): An osteological approach to the characterization of a relict wild population
Source: PLoS One. 2018 Apr 11;13(4):e0194727. doi: 10.1371/journal.pone.0194727 (PMC5894973; doi:10.1371/journal.pone.0194727)
Supplement: S1 Table — (DOCX) [file pone.0194727.s001.docx]

**S1 Table. Maximum weathering stages of the analyzed sample.**

| **Element** | **Code** | **Weathering (Adapted from Todd 1987)** | **Maximum stage** | **Chemical deposition** |
| --- | --- | --- | --- | --- |
| MR | MRCF15 | light cracking, no signs of flaking | 3 | no evidence |
| MR | MRCF16 | no signs of cracking or flaking | 1 | no evidence |
| MR | MRCF23 | light cracking, no signs of flaking | 3 | no evidence |
| MR | MRCF24 | light cracking, no signs of flaking | 3 | no evidence |
| MR | MRCF25 | no signs of cracking or flaking | 1 | no evidence |
| MR | MRMF1 | no signs of cracking or flaking | 1 | no evidence |
| MR | MRMF2 | no signs of cracking or flaking | 1 | Caco3 (<50%) |
| MX | CRNCF1 | moderate cracking, initial flaking in some areas | 4 | no evidence |
| MX | CRNCF10 | light cracking, no signs of flaking | 3 | no evidence |
| MX | CRNCF11 | Initial signs of cracking | 2 | no evidence |
| MX | CRNCF12 | moderate cracking, light flaking in some areas | 5 | no evidence |
| MX | CRNCF13 | moderate cracking, light flaking in some areas | 5 | no evidence |
| MX | CRNCF14 | Initial signs of cracking | 2 | no evidence |
| MX | CRNCF17 | moderate cracking, initial flaking in some areas | 4 | no evidence |
| MX | CRNCF18 | Initial signs of cracking | 2 | no evidence |
| MX | CRNCF19 | light cracking, no signs of flaking | 3 | no evidence |
| MX | CRNCF2 | moderate cracking, initial flaking in some areas | 4 | no evidence |
| MX | CRNCF20 | moderate cracking, initial flaking in some areas | 4 | no evidence |
| MX | CRNCF21 | light cracking, no signs of flaking | 3 | no evidence |
| MX | CRNCF3 | moderate cracking, initial flaking in some areas | 4 | no evidence |
| MX | CRNCF4 | light cracking, no signs of flaking | 3 | no evidence |
| MX | CRNCF5 | moderate cracking, initial flaking in some areas | 4 | no evidence |
| MX | CRNCF6 | moderate cracking, initial flaking in some areas | 4 | no evidence |
| MX | CRNCF7 | moderate cracking, initial flaking in some areas | 4 | no evidence |
| MX | CRNCF8 | moderate cracking, initial flaking in some areas | 4 | no evidence |
| MX | CRNCF9 | light cracking, no signs of flaking | 3 | no evidence |
| MX | CRNMF1 | no signs of cracking or flaking | 1 | no evidence |
| MX | CRNMF2 | Initial signs of cracking | 2 | no evidence |
| MX | CRNMF3 | no signs of cracking or flaking | 1 | no evidence |
| MX | CRNNO22 | no signs of cracking or flaking | 1 | no evidence |
| PHF | 443 | no signs of cracking or flaking | 1 | no evidence |
| PHF | 1357 | no signs of cracking or flaking | 1 | no evidence |
| PHF | 1535 | no signs of cracking or flaking | 1 | no evidence |
| PHF | 3810 | no signs of cracking or flaking | 1 | no evidence |
| PHF | 3871 | no signs of cracking or flaking | 1 | no evidence |
| PHF | 60-172 | no signs of cracking or flaking | 1 | no evidence |
| PHF | 62/2-1 | no signs of cracking or flaking | 1 | no evidence |
| PHF | AJPHF2 | no signs of cracking or flaking | 1 | no evidence |
| PHF | BPS191 | no signs of cracking or flaking | 1 | no evidence |
| PHF | BPS192 | no signs of cracking or flaking | 1 | no evidence |
| PHF | CF1 | no signs of cracking or flaking | 1 | no evidence |
| PHF | CF16 | no signs of cracking or flaking | 1 | no evidence |
| PHF | CF2 | no signs of cracking or flaking | 1 | no evidence |
| PHF | CF20 | no signs of cracking or flaking | 1 | no evidence |
| PHF | CF4 | no signs of cracking or flaking | 1 | no evidence |
| PHF | CF5 | no signs of cracking or flaking | 1 | no evidence |
| PHF | CF6 | no signs of cracking or flaking | 1 | no evidence |
| PHF | CF7 | no signs of cracking or flaking | 1 | no evidence |
| PHF | CF8 | no signs of cracking or flaking | 1 | no evidence |
| PHF | MF3 | no signs of cracking or flaking | 1 | no evidence |
